# F-box proteins: more than baits for the SCF?

**DOI:** 10.1186/1747-1028-1-30

**Published:** 2006-12-13

**Authors:** Damien Hermand

**Affiliations:** 1 Laboratoire de Génétique Moléculaire (GEMO), Facultés Universitaires Notre-Dame de la Paix, Rue de Bruxelles 61, 5000 Namur, Belgium

## Abstract

Regulation of protein stability through the ubiquitin proteasome system is a key mechanism underlying numerous cellular processes. The ubiquitin protein ligases (or E3) are in charge of substrate specificity and therefore play a pivotal role in the pathway. Among the several different E3 enzyme families, the SCF (Skp1-Cullin-F box protein) is one of the largest and best characterized. F-box proteins, in addition to the loosely conserved F-box motif that binds Skp1, often carry typical protein interaction domains and are proposed to recruit the substrate to the SCF complex. Strikingly, genomes analysis revealed the presence of large numbers of F-box proteins topping to nearly 700 predicted in *Arabidopsis thaliana*.

Recent evidences in various species suggest that some F-box proteins have functions not directly related to the SCF complex raising questions about the actual connection between the large F-box protein family and protein degradation, but also about their origins and evolution.

## Background

**Figure 1:**
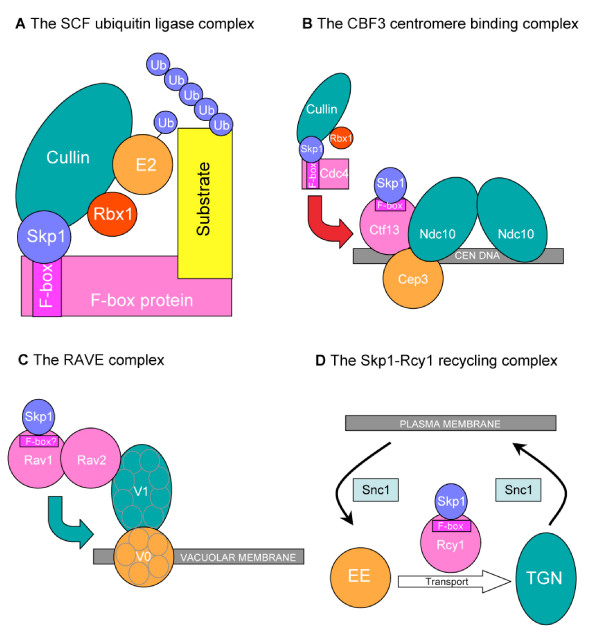
**F-box proteins that function with Skp1**. A. The SCF complex. The F-box protein is linked to the core complex through interaction between Skp1 and the F-box. Ubiquitin is transferred from a ubiquitin-conjugating enzyme (E2) onto substrates recruited by the F-box protein. After poly-ubiquitination, the substrate is recognized by the proteasome and degraded (not shown). B. The yeast CBF3 centromere binding complex. Activation of Ctf13 requires association with Skp1. This allows formation of the structural core of the budding yeast centromere binding complex (CBF) comprising Skp1, Ctf13, Cep3 and Ndc10. Ctf13 stability is controlled by ubiquitination dependent on a SCF complex containing the Cdc4 F-box protein. C. The RAVE complex. Skp1 forms the RAVE complex with Rav1 (containing a putative F-box motif) and Rav2. RAVE interacts with the V1 domain of the vacuolar membrane (H+)-ATPase (V-ATPase) and this is required for stable assembly of the V1 domain with the membrane associated V0 domain. Note that V1 and V0 are composed of several proteins. D. The Skp1-Rcy1 recycling complex. The Rcy1 F-box protein regulates the early endosomes (EE) to trans-golgi network (TGN) recycling. The Snc1 SNARE protein is endocytosed into early endosomes and is transported back to the plasma membrane via the trans-golgi network. Rcy1 binds Snc1 and regulate its sorting and marking for recycling by phosphorylation (not shown).

The small polypeptide ubiquitin (76 amino acids) is used in many processes including signal transduction, DNA repair, transcription and chromatin remodelling [1-5]. However, ubiquitin is best characterized has a post-translational modification required to label proteins for recognition and degradation by the proteasome [6]. Ubiquitin contains seven lysine residues used to form poly-ubiquitin chains and chains linked to lysine 48 are the most commonly used to target a substrate for proteolysis. Ubiquitin conjugation is catalysed by enzymes designated E1, E2 and E3. The E3 ubiquitin ligase determines substrate specificity. E3 complexes of the SCF subfamily typically contain at least four subunits: Skp1, a cullin, a RING finger protein (Rbx1, also called Hrt1 or Roc1), and a member of the large family of F-box adaptor protein directly involved in the substrates recruitment (for a review, see [7]). The F-box is a degenerated sequence of about 70 amino acids required but not sufficient for the interaction between a given F-box protein and Skp1 [8]. Structure-function studies in yeast and mammals have demonstrated that the cullin functions as a scaffold in assembling the different subunits of the SCF complex. The cullin interacts at its carboxyl terminus with the RING domain protein Rbx1 to form the catalytic domain, and at its amino terminus end with Skp1 (Figure 1A). Protein degradation mediated by the SCF complexes has been shown to influence a variety of cellular processes such as the cell cycle, signal transduction and gene expression [9-14]. Beside the canonical SCF, it appears that in some cases, Skp1 and F-box proteins may function in non-SCF complexes and that some F-box proteins have functions on their own. This commentary emphasizes these aspects.

## non SCF F-box protein-Skp1 complexes

In budding yeast, three Skp1-containing, non SCF complexes were described. The conserved CDEIII region (25 pb) in the budding yeast centromeric DNA is bound by CBF3, a protein complex containing p110 (Ndc10), p64 (Cep3), p58 (Ctf13) and Skp1 [15,16] (Figure 1B). CBF3 is a fundamental structure of the kinetochore and Skp1 plays a structural role for its assembly by activating the F-box protein Ctf13 [17]. The mechanism of activation is not well understood but is independent of SCF. Interestingly, Ctf13 is subject not only to positive regulation in a Skp1-dependent manner but also to negative regulation by degradation requiring a SCF complex comprising the F-box protein Cdc4 [18].

Skp1 also associates with Rav1 to form part of a complex termed RAVE (Regulator of the (H+)-ATPase of the Vacuolar and Endosomal membranes) (Figure 1C). RAVE is required to promote glucose-triggered assembly of the V-ATPase holoenzyme [19-21]. Although Rav1 is not classified as a *bona fide *F-box protein, its sequence harbours potential F-box motifs. Since other components of the SCF cannot be co-immunoprecipitated with RAVE, it is likely that RAVE also constitutes a non-SCF complex.

The F-box protein Rcy1 which is required for recycling of the SNARE (Soluble NSF Attachment protein REceptor) Snc1 from endosomes to the plasma membrane constitutes a third example of association between an F-box protein and Skp1 independently of SCF [22,23] (Figure 1D). In this case, the fission yeast homologue of Rcy1 (named Pof6) was also shown to form an *in vivo *complex with Skp1, independently of other SCF components [24].

In all of these cases, Skp1 is found to play an essential structural or activating role. Its associated F-box proteins interact with other proteins in a way reminiscent of the SCF but in the absence of the catalytic subunit, no ubiquitination and degradation occur.

## F-box proteins on their own

Recent work has demonstrated that some F-box proteins function independently of their F-box motif or their interaction with Skp1. In several cases however, association with Skp1 and the SCF is required to regulate the degradation of the F-box protein itself, or of other substrates. A good example of such a case is the Rca1/Emi1 F-box protein (Table 1) which is an essential inhibitor of the anaphase-promoting complex (APC-cyclosome) preventing premature cyclin degradation [25,26]. The F-box of Rca1 is dispensable for its inhibitory effect on the APC, yet Rca1 interacts with components of the SCF and its F-box is required for a second, uncharacterized function at the G1-S transition [25,27,28].

**Table 1:** Examples of F-box proteins that function independently of Skp1

F-box protein	Organism	Reference	Function
Rca1	*D. melanogaster*	25–28	Inhibition of Anaphase Promoting Complex in G2
Mfb1	*S. cerevisiae*	30, 31	Mitochondrial distribution and morphology
Mdm30	*S. cerevisiae*	30, 31	Mitochondrial distribution and morphology
Fbh1*	*H. sapiens, S. pombe*	33–36	Repression of recombination (DNA helicase)
MoKA	*M. musculus*	37	Transcription factor co-activator
Pof14	*S. pombe*	38	Repression of ergosterol synthesis

*in this case, it is unclear if the F-box is required *in vivo*. Note that some of the F-box proteins cited bind Skp1 or form an SCF complex but the function indicated is independent of this binding. Species included are *Saccharomyces cerevisiae, Schizosaccharomyces pombe, Drosophila melanogaster, Homo sapiens *and *Mus musculus*.

In budding yeast, two F-box proteins have non-redundant, F-box independent roles in mitochondrial morphology (Table 1). Mitochondria carry out key functions in the eukaryotic cells ranging from ATP supply generated by oxidative phosphorylations to assembly of the Fe/S clusters, or regulation of apoptosis [29]. The F-box proteins Mfb1 and Mdm30 are both required for proper mitochondrial distribution and morphology during mitotic growth. Mitochondria in strains deleted for Mfb1 are fusion competent but form aberrant interconnected tubules, while in the absence of Mdm30, they appear highly fragmented due to defects in fusion. These phenotypes cannot be mimicked by depletion of SCF core complex and F-box deleted versions of either Mfb1 or Mdm30 are competent to rescue them [30,31]. From these data, it is unclear what is the function of the F-box present in both proteins. One clue comes from the fact that Mfb1 preferentially localizes to mitochondria in the mother cell and this asymmetry requires its F-box [30], raising the possibility that Skp1 dependent degradation of Mfb1 occurs in the bud. This hypothesis is based on the fact that F-box proteins can be degraded autocatalytically in a ubiquitination – dependent fashion [32]. Further work is required to determine whether this is the case and how it is regulated.

The first F-box protein shown to possess intrinsic enzymatic activity is Fbh1, a DNA-dependent ATPase with a DNA unwinding activity displacing duplex DNA in the 3' to 5' direction [33]. In human cells, Fbh1 is part of a canonical SCF complex that retains the helicase and ATPase activities. However, these activities are indistinguishable from those of the Fbh1 protein alone (Table 1). Paradoxically, deletion of the F-box motif strongly reduced the enzymatic activities [34] although the deletion did not include any helicase motifs. Two studies addressed the role of the fission yeast homologue of Fbh1. On the first hand, they clarified the *in vivo *function of Fbh1 in processing toxic recombination intermediates. They also investigated the dependency of this function on an intact F-box motif. One study concluded that introduction of mutations in the F-box revealed a relatively minor role of the motif in Fbh1 function [35], while the second reports that mutation of the F-box mimics the deletion phenotype [36]. Clearly, more work is needed to clarify if Fbh1 is an active enzyme on its own *in vivo *and what is the role of the SCF complex it belongs to.

In two cases, F-box proteins have been shown to bind to and to modulate the activity of another protein independently of SCF and ubiquitination. Mammalian KLF (Krüppel-like factors) comprise transcription factors emerging as critical regulators of development. A two-hybrid screen with the KLF7 as bait identified the MoKA F-box protein (Table 1). MoKA turned out to function as a KLF7 co-activator regulating gene expression during cell differentiation [37]. The interaction region encompasses the F-box of MoKA, suggesting that it is independent of Skp1 and SCF. Supporting this, it was excluded that MoKA interaction may target KLF7 to the ubiquitination machinery [37].

In fission yeast, an interaction between the F-box protein Pof14 and the Erg9 squalene synthase, a key enzyme in ergosterol metabolism, was also recovered in a two-hybrid screen (Table 1). Pof14 binds to and inhibits Erg9 activity in response to oxidative stress independently of its F-box or other SCF components [38]. Deletion of *pof14 *leads to a marked decrease in viability in the presence of hydrogen peroxide, suggesting that inhibition of Erg9 and subsequent decrease in ergosterol level plays an important role in the adaptation to this stress. Pof14 also belongs to an SCF complex regulating its own level as reported for several other F-box proteins [32]. This keeps Pof14 at very low level in normal conditions, which raises the question of the physiological significance of this regulation. The case of Pof14 is revealing because deletion of the F-box leads to subtle defects in trafficking and sorting of proteins to the cell surface due to long-term decrease in ergosterol content. The rapid, SCF dependent, degradation of Pof14 after adaptation to oxidative stress is therefore essential to maintain a critical balance in ergosterol content. In the case of Pof14, the F-box motif works more as a destruction box in a way reminiscent of other degradation signals or degrons. An interesting possibility is that other members of the F-box family behave similarly.

## The fission yeast set of F-box proteins as mediator of environmental stress adaptation?

The availability of postgenomic data in fission yeast [39] allows us to examine the behaviour of the transcriptome representing the whole set of F-box proteins (which includes 18 members) in response to various stresses, versus the behaviour of the core SCF. From the dataset reported by Chen et al. [39], we looked at the transcriptional behaviour of the 18 members of the F-box family and the SCF core components Skp1 and Pcu1 in response to environmental stress (Table 2). Both SCF core components Skp1 and Pcu1 do not show consistent increase in transcription in response any of the tested stress. At the opposite, it clearly emerges that the majority of fission yeast F-box proteins are strongly induced by specific stresses (Table 2). Typical examples include Pof1 which is strongly induced by Cadmium, in line with recent data showing that SCF^Pof1 ^and its target Zip1 mediates cadmium response in fission yeast [40]; Pof4 induced by osmotic stress; or Pof14 induced in response to oxidative stress as mentioned above [38]. The only stress leading to a more global induction is heat shock, presumably because it is a very commonly encountered stress affecting a large number of cellular aspects.

**Table 2:** Induction of the *S. pombe *F-box proteins set in response to various stress

	H_2_O_2_	Cadmium	Heat	Sorbitol	MMS
Skp1	-	-	+	-	-
Pcu1	-	+	++	-	-
Pof1	++	+++	+	-	-
Pof2	ND	ND	ND	ND	ND
Pof3	-	-	-	-	-
Pof4	-	-	-	++	-
Pof5	-	+++	+++	-	-
Pof6	+	-	+	-	-
Pof7	-	-	+	-	-
Pof8	ND	ND	ND	ND	ND
Pof9	++	-	+	-	-
Pof10	-	-	+	-	-
Pof11	++	-	+	-	-
Pof12	-	-	+	-	-
Pof13	-	-	+++	-	-
Pof14	+++	+	++	-	-
Fbh1	-	-	-	-	-
Pop1	-	-	-	-	-
Pop2	-	-	-	-	-
SPAPB1a10.14	-	-	-	+	++

Fission yeast F-box proteins are induced by specific stresses. From the dataset generated by Chen *et al*. [39] and available from the Sanger centre web site [46], we selected all the predicted fission yeast F-box protein coding genes and the SCF core component coding genes *skp1 *and *pcu1*. They are classified in the following way: + : induction > 1,5 fold, ++ : induction > 2 fold, +++ : induction > 2,5 fold in response to indicated stress. H_2_O_2 _: hydrogen peroxide, MMS: methyl-methanesulfonate, ND: not determined.

In contrast, the F-box proteins Pop1 and Pop2 that play a constitutive role in normal cell cycle regulation [41-43], are not induced by stress. One can therefore envisage the intriguing possibility that a significant number of F-box proteins are intrinsically unstable proteins (through their association with SCF) primarily used for a specific function (as adaptation to the environment) that is independent of SCF. Beside their own function, they could also serve as adaptors between other proteins and the core ubiquitin ligase. With its limited set of F-box proteins and the ease of combining genetic and proteomic analyses, fission would be an ideal model to test this hypothesis.

## Conclusion and relevance

From about 20 F-box proteins in yeast to hundreds in higher eukaryotes, the F-box motif was used successfully during evolution. Proteins harbouring the F-box usually contain a wide range of other motifs including zinc fingers, leucine zipper, ring fingers, tetratricopeptide repeats (TPR) or proline rich regions. This diversity in the F-box protein family suggests that the F-box was included in these proteins as a second step. As mentioned above, one hypothesis is that the F-box motif was first used as a destruction motif. Subsequently, its role might have been extended to the recruitment of biochemical partners of F-box proteins to SCF, allowing their ubiquitination and degradation. This strategy couples an activity of the F-box protein (as the activation of an associated protein) to proteolytic degradation of either the partner alone or both the partner and the F-box protein. In many respects, this is reminiscent of the "unstable when active" phenomenon seen with many transcription factors [44]. Particularly illuminating is the case of the Skp2 F-box protein which enhances the activity of the transcription regulatory protein c-Myc, and at the same time promotes its ubiquitination and degradation when associated with the core SCF [45].

Taken together, the examples described here show that many variations are found on the F-box theme. They also indicate that the simple view that F-box proteins only serve as bait to recruit substrates to the core SCF should now be revised.

## Competing interests

The author(s) declare that they have no competing interests.

## References

[B1] Haglund K, Dikic I (2005). Ubiquitylation and cell signaling. Embo J.

[B2] Hicke L, Dunn R (2003). Regulation of membrane protein transport by ubiquitin and ubiquitin-binding proteins. Annu Rev Cell Dev Biol.

[B3] Zhang Y (2003). Transcriptional regulation by histone ubiquitination and deubiquitination. Genes Dev.

[B4] Huang TT, D'Andrea AD (2006). Regulation of DNA repair by ubiquitylation. Nat Rev Mol Cell Biol.

[B5] Dhananjayan SC, Ismail A, Nawaz Z (2005). Ubiquitin and control of transcription. Essays Biochem.

[B6] Hershko A, Ciechanover A (1998). The ubiquitin system. Annu Rev Biochem.

[B7] Weissman AM (2001). Themes and variations on ubiquitylation. Nat Rev Mol Cell Biol.

[B8] Schulman BA, Carrano AC, Jeffrey PD, Bowen Z, Kinnucan ER, Finnin MS, Elledge SJ, Harper JW, Pagano M, Pavletich NP (2000). Insights into SCF ubiquitin ligases from the structure of the Skp1-Skp2 complex. Nature.

[B9] Bai C, Sen P, Hofmann K, Ma L, Goebl M, Harper JW, Elledge SJ (1996). SKP1 connects cell cycle regulators to the ubiquitin proteolysis machinery through a novel motif, the F-box. Cell.

[B10] Mathias N, Johnson S, Byers B, Goebl M (1999). The abundance of cell cycle regulatory protein Cdc4p is controlled by interactions between its F box and Skp1p. Mol Cell Biol.

[B11] Willems AR, Lanker S, Patton EE, Craig KL, Nason TF, Mathias N, Kobayashi R, Wittenberg C, Tyers M (1996). Cdc53 targets phosphorylated G1 cyclins for degradation by the ubiquitin proteolytic pathway. Cell.

[B12] Feldman RM, Correll CC, Kaplan KB, Deshaies RJ (1997). A complex of Cdc4p, Skp1p, and Cdc53p/cullin catalyzes ubiquitination of the phosphorylated CDK inhibitor Sic1p. Cell.

[B13] Skowyra D, Craig KL, Tyers M, Elledge SJ, Harper JW (1997). F-box proteins are receptors that recruit phosphorylated substrates to the SCF ubiquitin-ligase complex. Cell.

[B14] Skowyra D, Koepp DM, Kamura T, Conrad MN, Conaway RC, Conaway JW, Elledge SJ, Harper JW (1999). Reconstitution of G1 cyclin ubiquitination with complexes containing SCFGrr1 and Rbx1. Science.

[B15] Lechner J, Ortiz J (1996). The Saccharomyces cerevisiae kinetochore. FEBS Lett.

[B16] Bouck D, Bloom K (2005). The role of centromere-binding factor 3 (CBF3) in spindle stability, cytokinesis, and kinetochore attachment. Biochem Cell Biol.

[B17] Kitagawa K, Skowyra D, Elledge SJ, Harper JW, Hieter P (1999). SGT1 encodes an essential component of the yeast kinetochore assembly pathway and a novel subunit of the SCF ubiquitin ligase complex. Mol Cell.

[B18] Kaplan KB, Hyman AA, Sorger PK (1997). Regulating the yeast kinetochore by ubiquitin-dependent degradation and Skp1p-mediated phosphorylation. Cell.

[B19] Smardon AM, Tarsio M, Kane PM (2002). The RAVE complex is essential for stable assembly of the yeast V-ATPase. J Biol Chem.

[B20] Kane PM, Smardon AM (2003). Assembly and regulation of the yeast vacuolar H+-ATPase. J Bioenerg Biomembr.

[B21] Seol JH, Shevchenko A, Shevchenko A, Deshaies RJ (2001). Skp1 forms multiple protein complexes, including RAVE, a regulator of V-ATPase assembly. Nat Cell Biol.

[B22] Galan JM, Wiederkehr A, Seol JH, Haguenauer-Tsapis R, Deshaies RJ, Riezman H, Peter M (2001). Skp1p and the F-box protein Rcy1p form a non-SCF complex involved in recycling of the SNARE Snc1p in yeast. Mol Cell Biol.

[B23] Wiederkehr A, Avaro S, Prescianotto-Baschong C, Haguenauer-Tsapis R, Riezman H (2000). The F-box protein Rcy1p is involved in endocytic membrane traffic and recycling out of an early endosome in Saccharomyces cerevisiae. J Cell Biol.

[B24] Hermand D, Bamps S, Tafforeau L, Vandenhaute J, Makela TP (2003). Skp1 and the F-box protein Pof6 are essential for cell separation in fission yeast. J Biol Chem.

[B25] Reimann JD, Freed E, Hsu JY, Kramer ER, Peters JM, Jackson PK (2001). Emi1 is a mitotic regulator that interacts with Cdc20 and inhibits the anaphase promoting complex. Cell.

[B26] Schmidt A, Rauh NR, Nigg EA, Mayer TU (2006). Cytostatic factor: an activity that puts the cell cycle on hold. J Cell Sci.

[B27] Zielke N, Querings S, Grosskortenhaus R, Reis T, Sprenger F (2006). Molecular dissection of the APC/C inhibitor Rca1 shows a novel F-box-dependent function. EMBO Rep.

[B28] Schmidt A, Duncan PI, Rauh NR, Sauer G, Fry AM, Nigg EA, Mayer TU (2005). Xenopus polo-like kinase Plx1 regulates XErp1, a novel inhibitor of APC/C activity. Genes Dev.

[B29] Scheffler IE (2000). A century of mitochondrial research: achievements and perspectives. Mitochondrion.

[B30] Kondo-Okamoto N, Ohkuni K, Kitagawa K, McCaffery JM, Shaw JM, Okamoto K (2006). The novel F-box protein Mfb1p regulates mitochondrial connectivity and exhibits asymmetric localization in yeast. Mol Biol Cell.

[B31] Durr M, Escobar-Henriques M, Merz S, Geimer S, Langer T, Westermann B (2006). Nonredundant roles of mitochondria-associated F-box proteins Mfb1 and Mdm30 in maintenance of mitochondrial morphology in yeast. Mol Biol Cell.

[B32] Galan JM, Peter M (1999). Ubiquitin-dependent degradation of multiple F-box proteins by an autocatalytic mechanism. Proc Natl Acad Sci U S A.

[B33] Kim J, Kim JH, Lee SH, Kim DH, Kang HY, Bae SH, Pan ZQ, Seo YS (2002). The novel human DNA helicase hFBH1 is an F-box protein. J Biol Chem.

[B34] Kim JH, Kim J, Kim DH, Ryu GH, Bae SH, Seo YS (2004). SCFhFBH1 can act as helicase and E3 ubiquitin ligase. Nucleic Acids Res.

[B35] Osman F, Dixon J, Barr AR, Whitby MC (2005). The F-Box DNA helicase Fbh1 prevents Rhp51-dependent recombination without mediator proteins. Mol Cell Biol.

[B36] Morishita T, Furukawa F, Sakaguchi C, Toda T, Carr AM, Iwasaki H, Shinagawa H (2005). Role of the Schizosaccharomyces pombe F-Box DNA helicase in processing recombination intermediates. Mol Cell Biol.

[B37] Smaldone S, Laub F, Else C, Dragomir C, Ramirez F (2004). Identification of MoKA, a novel F-box protein that modulates Kruppel-like transcription factor 7 activity. Mol Cell Biol.

[B38] Tafforeau L, Le Blastier S, Bamps S, Dewez M, Vandenhaute J, Hermand D (2006). Repression of ergosterol level during oxidative stress by fission yeast F-box protein Pof14 independently of SCF. Embo J.

[B39] Chen D, Toone WM, Mata J, Lyne R, Burns G, Kivinen K, Brazma A, Jones N, Bahler J (2003). Global transcriptional responses of fission yeast to environmental stress. Mol Biol Cell.

[B40] Harrison C, Katayama S, Dhut S, Chen D, Jones N, Bahler J, Toda T (2005). SCF(Pof1)-ubiquitin and its target Zip1 transcription factor mediate cadmium response in fission yeast. Embo J.

[B41] Kominami K, Toda T (1997). Fission yeast WD-repeat protein pop1 regulates genome ploidy through ubiquitin-proteasome-mediated degradation of the CDK inhibitor Rum1 and the S-phase initiator Cdc18. Genes Dev.

[B42] Kominami K, Ochotorena I, Toda T (1998). Two F-box/WD-repeat proteins Pop1 and Pop2 form hetero- and homo-complexes together with cullin-1 in the fission yeast SCF (Skp1-Cullin-1-F-box) ubiquitin ligase. Genes Cells.

[B43] Wolf DA, McKeon F, Jackson PK (1999). F-box/WD-repeat proteins pop1p and Sud1p/Pop2p form complexes that bind and direct the proteolysis of cdc18p. Curr Biol.

[B44] Muratani M, Tansey WP (2003). How the ubiquitin-proteasome system controls transcription. Nat Rev Mol Cell Biol.

[B45] von der Lehr N, Johansson S, Wu S, Bahram F, Castell A, Cetinkaya C, Hydbring P, Weidung I, Nakayama K, Nakayama KI, Soderberg O, Kerppola TK, Larsson LG (2003). The F-box protein Skp2 participates in c-Myc proteosomal degradation and acts as a cofactor for c-Myc-regulated transcription. Mol Cell.

[B46] http://www.sanger.ac.uk/PostGenomics/S_pombe/

